# Changes in retinal flow density measured by optical coherence tomography angiography in patients with carotid artery stenosis after carotid endarterectomy

**DOI:** 10.1038/s41598-018-35556-4

**Published:** 2018-11-21

**Authors:** Larissa Lahme, Elena Marchiori, Giuseppe Panuccio, Pieter Nelis, Friederike Schubert, Natasa Mihailovic, Giovanni Torsello, Nicole Eter, Maged Alnawaiseh

**Affiliations:** 1 Department of Ophthalmology, University of Muenster Medical Center, Muenster, Germany; 2 Department of Vascular and Endovascular Surgery, University of Muenster, Muenster, Germany

## Abstract

The aim of the study presented here was to evaluate retinal and optic nerve head (ONH) perfusion in patients with severe asymptomatic carotid artery stenosis (CAS) compared with healthy controls and to analyze the impact of carotid endarterectomy using optical coherence tomography angiography (OCT-A). 25 eyes of 25 patients with CAS (study group) and 25 eyes of 25 healthy controls (control group) were prospectively included in this study. OCT-A was performed using RTVue XR Avanti (Optovue, Inc, Fremont, California, USA). The flow density data in the superficial and deep retinal OCT-angiogram of the macula and in the radial peripapillary capillary network (RPC) of the ONH were extracted and analyzed. The flow density in the superficial retinal OCT angiogram of the macula and in the ONH were significantly lower in the study group compared with the control group (macula: p = 0.003) (ONH: p = 0.013). The flow density in the ONH improved significantly after carotid endarterectomy (p = 0.004). A reduced flow density was observed in patients with CAS when compared with healthy controls. The flow density also improved after carotid endarterectomy. Quantitative changes in the microvascular density, as measured using OCT-A, could well be useful in the diagnosis of CAS and the evaluation of therapy success.

## Introduction

Carotid artery stenosis is an important risk factor for ischemic stroke and transient ischemic attacks^1^. Management of vascular risk factors, antiplatelet therapy and different surgical procedures (carotid endarterectomy, carotid angioplasty and stenting) are therapeutic options available for the management of carotid artery stenosis. Detection of stenosis of the carotid artery is therefore especially important in neurologically asymptomatic patients^2–4^. In current guidelines, the degree of stenosis is an important surrogate measure for stroke risk and indication for intervention. Various imaging technologies such as CT angiography, magnetic resonance angiography and/or duplex ultrasonography are therefore used to evaluate patients with CAS^5,6^.

Internal carotid artery stenosis can be associated with impaired ocular blood flow and retinal examination is generally performed in internal carotid artery stenosis patients when clinical ocular symptoms such as sudden or progressive visual loss occur^7^. However, chronic carotid artery stenosis is not necessarily associated with morphological or functional retinal damage^7^.

Optical coherence tomography angiography (OCT-A) is a novel technology, providing high-resolution images of the retinal vasculature^8^. This method also enables quantitative evaluation of the retinal blood flow and blood flow in the optic nerve head (ONH)^9–11^.

The aim of the study presented here was to evaluate the retinal and optic nerve head (ONH) perfusion, as measured using optical coherence tomography angiography (OCT-A) in patients with asymptomatic severe internal carotid artery stenosis compared to healthy controls, and to evaluate the impact of carotid endarterectomy.

## Methods

### Patients

For this prospective study 25 eyes of 25 patients suffering from internal carotid artery stenosis were consecutively enrolled. A control group of 25 eyes of 25 healthy subjects without ocular disease of any sort were also included. 18 patients were planned for surgical treatment and were evaluated before and after surgery. The study followed the tenets of the Declaration of Helsinki and was approved by the Ethics Committee of the University of Muenster, North Rhine Westphalia, Germany. Before imaging, the study protocol was explained in detail and all participants signed an informed consent form.

Inclusion criteria were asymptomatic patients with carotid artery stenosis ≥70% with perioperative risk <3% (Guidelines for the primary prevention of stroke of the American Heart Association/American Stroke Association^12^), age over 18 years, and a planned surgical treatment.

Exclusion criteria were ocular symptoms (visual loss or visual impairment), media opacities preventing high-quality imaging, vitreoretinal disease or status post vitreoretinal surgery. Patients with neurological diseases, myocardial infarction or strokes were excluded. Patients with diabetes without diabetic retinopathy were included. The comorbidities of the study group and control group are summarized in Table 1 (Table 1).

**Table 1:** Clinical characteristics of the study groups.

	Study group	Control group	p-Value
	mean ± SD	mean ± SD	
**n**	25	25	
**age (years)**	64.56 ± 7.23	64.76 ± 9.81	0.935
**sphericalequivalent (D)**	0.81 ± 1.16	0.52 ± 1.47	0.503
**IOD**	13.95 ± 2.39	15.25 ± 2.49	0.136
**visualacuity**	0.86 ± 0.17	0.89 ± 0.18	0.604
**comorbidity**			
diabetes	2	2	
art. hypertension	21	7	
hyperlipoproteinemia	21	3	

Bold: statistically significant results.

Before imaging subjects were also asked to take a rest of about 5 minutes and systemic blood pressure was measured in the left brachial artery at the height of the heart with the subject in an upright sitting position. Subjects with systolic blood pressure (>150 mmHg/<100 mmHg) or diastolic blood pressure (>90 mmHg/ <60 mmHg) were not included.

### Surgical Treatment

Surgical treatment was performed in the Department of Vascular Surgery at the University of Muenster Medical Center. Surgery was carried out under general anesthesia. After exposure of the carotid bifurcation and administration of a bolus of heparin, the internal, common and external carotid arteries were clamped. A shunt was placed to maintain perfusion of the intracranial vessels during the procedure and a meticulous removal of the plaque was performed. Depending on the anatomical characteristics of the bifurcation, an eversion endarterectomy with direct suture or a longitudinal endarterectomy with Dacron patch was carried out. All patients were postoperatively monitored in an intermediate care ward for at least 24 hours.

### Examination

Before OCT-A imaging, patients underwent a complete ocular examination including refraction, IOP (intraocular pressure) measurement, slit lamp biomicroscopy and funduscopy. OCT-A imaging was performed before and 3–4 days after surgery. OCT-A imaging was performed using the AngioVue device (RTVue XR Avanti with AngioVue, OptovueInc, Fremont, California, USA). This system has an A-scan rate of 70,000 scans/second and the split-spectrum amplitude-decorrelation angiography (SSADA) algorithm was used to generate the angiography data. The OCT-A technology has been described in detail elsewhere^9,11^. Briefly, repeated OCT scans of a certain area are performed and the OCT images of that area evaluated to identify possible changes. Blood flow in the retinal vessels will result in changes between the successive OCT images, whereas static tissue will show no change^11^.

OCT-A imaging was performed in the same location by an expert examiner under the same conditions. Imaging of the optic nerve head (ONH) required a 4.5 × 4.5 mm^2^ scans while macula imaging required a 3.0 × 3.0 mm^2^ scan. Images of poor quality (lines or gaps arising from poor signal strength or motion artifacts) were excluded from the study. The software automatically segmented the tissue into 4 layers: in the ONH (optic nerve head, vitreous, radial peripapillary capillary (RPC), and choroid) and in the macula (superficial, deep, outer retina and choriocapillaris). After checking the segmentations, the flow density data in the optic nerve head (radial peripapillary capillary (RPC) layer and the macula (superficial and deep retinal OCT angiogram) were then extracted and analyzed.

### Data analysis and statistics

Data management was performed using Microsoft Excel 2013. IBM SPSS® Statistics 22 for Windows (IBM Corporation, Somers, NY, USA) was used for statistical analyses. The normality of the data distribution was tested using the Kolmogorov–Smirnov test. After confirmation of the normality assumption data are generally presented as mean ± standard deviation while changes at follow-up compared with baseline were assessed using paired sample t-tests. The two treatment groups were compared using independent Student’s t-tests. All inferential statistics are intended to be exploratory, not confirmatory, and are interpreted accordingly. The global statistical significance level was set to 0.05.

## Results

25 patients with CAS (age: 64.56 ± 7.23) and 25 healthy control subjects (age: 64.76 ± 9.81) were prospectively included in the study. There was no statistically significant difference in age between the two groups (p = 0.94). Clinical characteristics of the study group and the control group are summarized in Table 1.

There was no significant difference between the signal strength indices (SSI) in the control group and the study group (SSI of the macula measurements: study group: 69.23 ± 7.57; control group: 69.81 ± 6.27; p = 0.78; SSI of the ONH measurements: study group: 63.78 ± 7.84, control group: 68.18 ± 4.32, p = 0.07).

**Figure 1:**
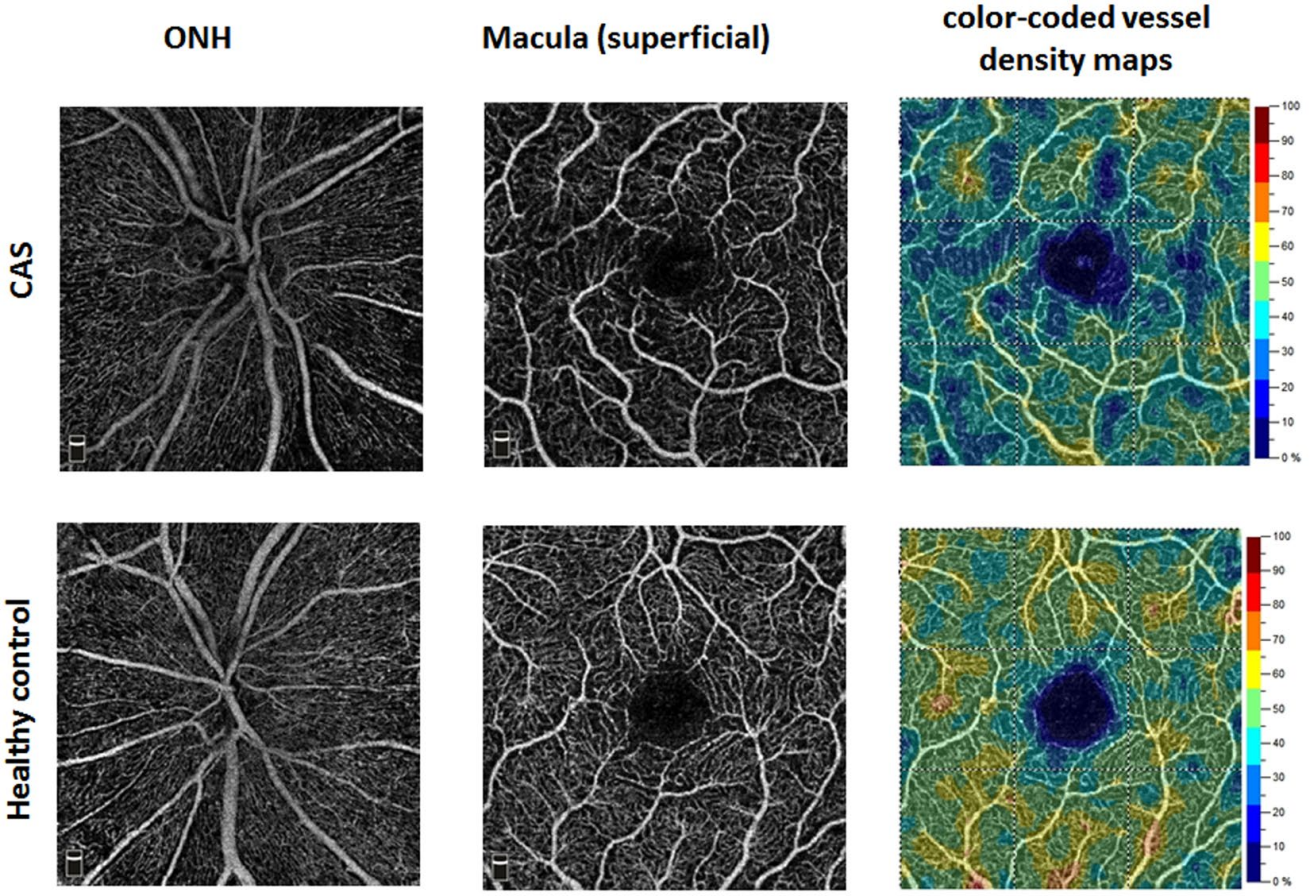
OCT angiograms of a patient with CAS (Top row) and a healthy control (Bottom row).

The flow density (whole *en face*) in the superficial retinal OCT angiogram of the macula in patients with CAS was significantly lower compared with healthy controls (study group: 48.52 ± 4.46; control group: 51.88 ± 2.70; p = 0.003) (Fig. 1). Significant differences were also found in the ONH. The flow density data in the macula and ONH of the study group and the control group are summarized in Table 2.

**Table 2:** Flow density in the macula and optic nerve head of patients with CAS and of healthy controls.

	Study group	Control group	p-Value
	mean ± SD	mean ± SD	
**OCT-A superficial**			
whole en face	48.52 ± 4.46	51.88 ± 2.70	**0.003**
fovea	28.31 ± 7.95	28.84 ± 6.04	0.726
parafovea	50.72 ± 4.39	54.08 ± 2.57	**0.002**
**OCT-A deep**			
whole en face	54.88 ± 4.44	55.07 ± 4.77	0.892
fovea	32.79 ± 8.31	31.81 ± 9.31	0.706
parafovea	57.03 ± 4.73	57.05 ± 5.41	0.989
**OCT-A RPC**			
whole en face	51.53 ± 3.72	54.17 ± 3.43	**0.013**
inside Disc	40.02 ± 9.12	47.88 ± 6.61	**<0.001**
peripapillary	58.96 ± 6.10	60.43 ± 5.43	0.376

Bold: statistically significant differences between the two groups.

In patients with CAS there were no significant differences in flow density between the ipsilateral and the contralateral eye at baseline (p > 0.05). The flow density (whole *en face*) in the RPC in the ipsilateral eyes, improved significantly after carotid endarterectomy. The flow density values in the ipsilateral eyes are summarized in Table 3 and the flow density values of the contralateral eyes in Table 4.

**Table 3:** Flow density values obtained in the indicated regions in the ipsilateral eyes before and after carotid endarterectomy.

n = 18	preopertive	postoperative	Relative change (%)	p-Value
	mean ± SD	mean ± SD		
**OCT-A superficial**				
whole en face	50.21 ± 2.23	50.38 ± 2.32	0.34	0.720
fovea	30.82 ± 6.21	29.50 ± 5.36	−4.28	0.238
parafovea	52.25 ± 2.69	52.29 ± 2.62	0.08	0.857
**OCT-A deep**				
whole en face	56.84 ± 2.12	56.57 ± 2.09	−0.48	0.590
fovea	33.58 ± 8.36	32.13 ± 5.81	−4.32	0.399
parafovea	59.08 ± 2.46	58.60 ± 2.63	−0,81	0.483
**OCT-A RPC**				
whole en face	53.06 ± 2.69	54.59 ± 2.39	2.88	**0.004**
inside Disc	37.58 ± 8.60	39.03 ± 8.16	3.86	0.265
peripapillary	62.21 ± 2.12	63.62 ± 2.43	2.27	**0.005**

Bold: statistically significant differences.

**Table 4:** Flow density values obtained in the indicated regions in the contralateral eyes before and after carotid endarterectomy.

n = 18	preopertive	postoperative	Relative change (%)	p-Value
	mean ± SD	mean ± SD		
**OCT-A superficial**				
whole en face	49.16 ± 4.28	49.53 ± 3.02	0.75	0.775
fovea	29.65 ± 5.74	29.89 ± 5.20	0.81	0.733
parafovea	51.15 ± 4.31	51.36 ± 3.37	0.41	0.847
**OCT-A deep**				
whole en face	55.28 ± 3.44	55.61 ± 4.41	0.60	0.956
fovea	32.17 ± 5.28	32.38 ± 5.57	0.65	0.578
parafovea	57.35 ± 3.34	57.66 ± 4.47	0.54	0.991
**OCT-A RPC**				
whole en face	52.69 ± 3.86	54.54 ± 3.30	3.51	**0.004**
inside Disc	36.87 ± 9.72	38.86 ± 7.26	5.40	0.233
peripapillary	62.22 ± 3.18	63.90 ± 3.29	2.70	**0.003**

Bold: statistically significant differences.

## Discussion

This pilot study is the first to determine reduced flow density in patients with CAS compared with healthy controls using OCT-A. OCT-A is non-invasive and can be performed easily and fast. It enables visualization of blood flow in the retina and ONH without intravenously injected dye. This technology has attracted a great deal of clinical research interest over the last two years and is finding increasing use in clinical practice^9^. OCTA also enables quantitative analysis of flow density in the retina and optic nerve head and has been assessed in various ocular and systemic diseases^9,10,13–16^. The reproducibility of the quantitative analysis of flow density has been evaluated in healthy subjects and in patients with different ocular diseases^9,10,14^.

Various studies in the literature have evaluated morphological and functional ophthalmological parameters in patients with CAS compared with healthy controls: Sayin *et al*. found a decreased choroidal thickness in patients with CAS, while in the same study no significant difference was found in the thicknesses of the retinal nerve fiber layer, macula or ganglion cell complex^17^. Whereas Heßler *et al*. also failed to find a significantly reduced RNFL thickness in patients with CAS^7^, a community-based study recently published by Wang *et al*. does describe reduced RNFL thicknesses in patients with CAS^4^. In functional tests, Kofoed *et al*. described significantly reduced and delayed electroretinographic responses in patients with carotid artery stenosis^18^.

Stenosis of the carotid artery leads to a fall in ocular blood flow^7,19–21^. In our study, patients with CAS showed a reduced flow density in the RPC layer of the ONH and in the superficial retinal OCT-angiogram when compared with healthy controls. In the superficial retinal OCT-angiogram the differences in the parafovea and in the entire evaluated area (whole en face) were significant whereas the difference in the fovea did not reach the significance level. This could be explained by interindividual variation in the area of the FAZ (higher SD in the fovea when compared with parafovea)^22^. The difference between the two groups in the deep retinal OCT-angiogram was also not significant. However, the analysis of flow density values in the deep retinal OCT-angiogram should be interpreted with caution, since the quantification of flow density in the deep retinal OCT angiogram is more challenging, being affected by projection artefacts, and repeatability was found to be weaker compared with that of the superficial retinal OCT-angiogram in previous studies^11,23–25^.

Most of the patients included in our study had a bilateral CAS. Therefore there were no significant differences between the flow density of the ipsilateral eye and the contralateral eye at baseline. After carotid endarterectomy the flow density improved significantly in the ipsilateral and in the contralateral eye. Carotid revascularization surgery improves cerebral perfusion and has a positive effect on the contralateral cerebral blood flow through the collateral circulation^26–28^. The positive effect of carotid endarterectomy on the ipsilateral retinal blood flow has been reported before^21^. Lareyre *et al*. also demonstrated bilaterally increased choroidal thickness using enhanced depth imaging optical coherence tomography (EDI-OCT) in patients with CAS after carotid endarterectomy^26^. Lareyre *et al*. hypothesized that EDI OCT could be a potential marker for the assessment of cerebral and/or ocular perfusion after carotid endarterectomy^26^. An important issue to consider in this context is that collateral pathways through the ophthalmic artery may be recruited to compensate for diminished cerebral blood flow in patients with internal carotid artery stenosis^5^. Although OCT-A is fast, non-invasive, accurate and reproducible an important limitation of OCT-A that should be mentioned here is the absence of flow direction.

This pilot study is also limited by the small sample size and short follow-up period. Further studies with a larger number of patients and a longer follow-up period are now required to evaluate whether OCT-A could be useful as a potential marker in the diagnosis of CAS and the evaluation of treatment success.

To conclude patients with CAS showed a reduced flow density in the RPC layer (ONH) and in the superficial retinal OCT-angiogram when compared with healthy controls. The flow density improved significantly after surgical treatment (carotid endarterectomy). Measurement of OCT-A and quantitative analyses of flow density could represent a useful, fast, non-invasive and objective approach to diagnosis of CAS and evaluation of treatment success.

## Data Availability

The corresponding author had full access to all the data in the study and all authors shared final responsibility for the decision to submit for publication.
